# Facile Synthesis of Fe_3_O_4_@Au/PPy-DOX Nanoplatform with Enhanced Glutathione Depletion and Controllable Drug Delivery for Enhanced Cancer Therapeutic Efficacy

**DOI:** 10.3390/molecules27134003

**Published:** 2022-06-22

**Authors:** Chunxia Qi, Wanni Wang, Peisan Wang, Hanlong Cheng, Xueyan Wang, Baoyou Gong, Anjian Xie, Yuhua Shen

**Affiliations:** 1 College of Chemistry and Chemical Engineering, Anhui University, Hefei 230601, China; qicx666@mail.ustc.edu.cn (C.Q.); cheng19941125@163.com (H.C.); 1344145610@163.com (X.W.); 1010675402@163.com (B.G.); anjx@163.com (A.X.); 2 Department of Chemical and Chemical Engineering, Hefei Normal University, Hefei 230601, China; 3 School of Biomedical Engineering, Research and Engineering Center of Biomedical Materials, Anhui Medical University, Hefei 230032, China; wnwang@ahmu.edu.cn

**Keywords:** Fe_3_O_4_@Au/PPy nanoplatform, tumor microenvironment, glutathione-depleting, multimodal cancer therapy, DOX

## Abstract

The complex physiological environment and inherent self-healing function of tumors make it difficult to eliminate malignant tumors by single therapy. In order to enhance the efficacy of antitumor therapy, it is significant and challenging to realize multi-mode combination therapy by utilizing/improving the adverse factors of the tumor microenvironment (TME). In this study, a novel Fe_3_O_4_@Au/PPy nanoplatform loaded with a chemotherapy drug (DOX) and responsive to TME, near-infrared (NIR) laser and magnetic field was designed for the combination enhancement of eliminating the tumor. The Fe^2+^ released at the low pH in TME can react with endogenous H_2_O_2_ to induce toxic hydroxyl radicals (·OH) for chemodynamic therapy (CDT). At the same time, the generated Fe^3+^ could deplete overexpressed glutathione (GSH) at the tumor site to prevent reactive oxygen species (ROS) from being restored while producing Fe^2+^ for CDT. The designed Fe_3_O_4_@Au/PPy nanoplatform had high photothermal (PT) conversion efficiency and photodynamic therapy (PDT) performance under NIR light excitation, which can promote CDT efficiency and produce more toxic ROS. To maximize the cancer-killing efficiency, the nanoplatform can be successfully loaded with the chemotherapeutic drug DOX, which can be efficiently released under NIR excitation and induction of slight acidity at the tumor site. In addition, the nanoplatform also possessed high saturation magnetization (20 emu/g), indicating a potential magnetic targeting function. In vivo and in vitro results identified that the Fe_3_O_4_@Au/PPy-DOX nanoplatform had good biocompatibility and magnetic-targeted synergetic CDT/PDT/PTT/chemotherapy antitumor effects, which were much better than those of the corresponding mono/bi/tri-therapies. This work provides a new approach for designing intelligent TME-mediated nanoplatforms for synergistically enhancing tumor therapy.

## 1. Introduction

According to the latest estimated data from the World Health Organization’s International Agency for Research on Cancer (IARC), by 2020, the number of new cancer cases worldwide has risen to 19.29 million, and the number of deaths will reach 9.96 million. Malignant tumors have become one of the serious diseases threatening human health. Recently, more and more researchers are engaged in the design of multi-mode tumor therapy and the synthesis of related materials because the use of a single modality for cancer treatment is limited. Currently, chemotherapy/photodynamic therapy chemotherapy/photodynamic therapy (CT/PDT) [1,2], PDT/photothermal therapy (PDT/PTT) [3], CT/chemodynamic therapy (CT/CDT) [4], PDT/CDT [5] and other combination therapies improve the therapeutic effect because of their limited side effects and high drug sensitivity. However, the complex tumor microenvironment (TME), including factors such as low pH, hypoxia, H_2_O_2_ and glutathione (GSH) overexpression due to abnormal metabolism in malignant tumors [6,7,8], often weakens the antitumor effect. Therefore, it is very important to exploit these features of the TME to develop a multimodal treatment strategy with TME synergistic responsiveness.

With the development of nanomedicine, CDT has received increasing attention due to its specific activation mechanism in the TME [9,10]. CDT relies on the Fenton/Fenton-like activity between the less active hydrogen peroxide (H_2_O_2_) and Fe, Cu, Co or Mn-based reagents to generate the most cytotoxic reactive oxygen species (ROS), hydroxyl radicals (OH), inducing apoptosis and necrosis in cancer cells [11,12,13]. CDT can greatly avoid damage to normal cells/tissues by exploiting in situ tumor-site-specific intracellular production of cytotoxic free radicals. However, the toxic ROS produced by CDT or other therapeutic approaches can be easily eliminated by the overexpressed GSH of the TME, resulting in a weakened therapeutic effect [14,15]. In order to improve CDT efficiency and increase ROS production, several strategies can be considered, such as the selection of appropriate nanomaterials and the adjustment of the reaction environment (increasing local temperature, decreasing pH, increasing reactant amount, decreasing glutathione amount) [16,17]. In addition, in order to further increase ROS production, PDT and CDT combination therapy should be considered. Meanwhile, PTT can improve tumor local temperature, promote the Fenton reaction to overcome activation barriers and increase ROS production. Therefore, it is of great significance to develop TME-responsive nanoplatforms for CDT/PDT/PTT combination therapy for tumor treatment.

Fe_3_O_4_ nanoparticles (NPs) are not only the preferred materials for magnetic targeting due to their high saturation magnetization [18,19,20,21,22,23], but also the focus of research on their chemical kinetic properties [24,25]. Fe_3_O_4_ NPs have good internal stability and have been shown to be non-cytotoxic at neutral pH [26]. Under the slightly acidic conditions of the tumor, they can self-sacrifice to produce Fe^2+^ and catalyze H_2_O_2_ to produce •OH through the Fenton reaction, thus killing cancer cells. Meanwhile, Fe^3+^ ions have multi-TME-response characteristics; they can consume overexpressed GSH in tumor cells to prevent ROS recovery and generate Fe^2+^ for CDT. At present, the widely used photothermal agents mainly include noble metal nanomaterials (Au [27,28], Ag [29]), carbon nanomaterials (carbon nanotube [30,31,32] and graphene [33,34]) and conjugated polymers [35,36]. As photothermal reagents, polypyrrole (PPy) and Au NPs can inhibit tumor growth and promote CDT efficiency of Fe_3_O_4_ NPs by generating local heat under the excitation of NIR light. On the other hand, Au and PPy can form a FRET system. Through the surface plasmon resonance (SPR) effect, Au NPs can effectively absorb NIR light and transfer the absorbed light energy to PPy to produce more ROS. Therefore, the combination of these three treatments to construct a dual-response (TME- and NIR-responsive) nanoplatform can reduce the antioxidant capacity of the tumor and improve the efficiency of CDT and ROS generation, thus having synergistic antitumor potential.

**Scheme 1:**
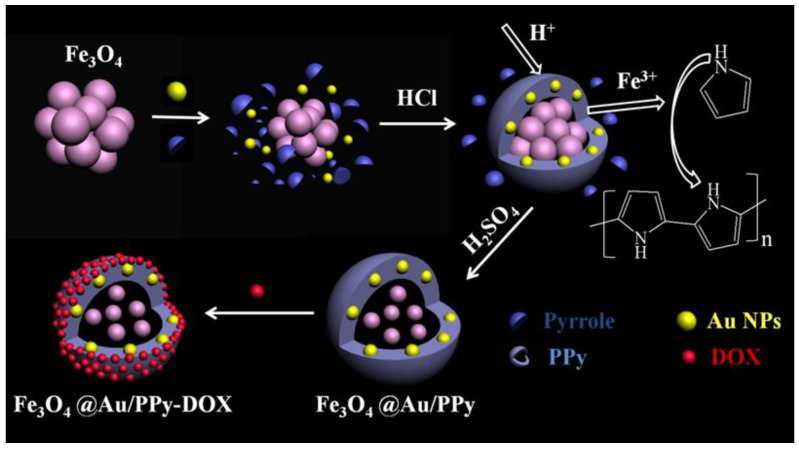
Illustration of the fabrication of the Fe_3_O_4_@Au/PPy nanoplatform.

Here, we prepared a multifunctional core@shell nanoplatform (Fe_3_O_4_@Au/PPy) with magnetic Fe_3_O_4_ NPs as cores and PPy and Au NPs as shells using a self-sacrificial templating method (Scheme 1). Fe_3_O_4_ can slowly release Fe^2+^ and Fe^3+^ under the induction of the low-pH environment of tumor cells. Fe^2+^ can induce a CDT effect with endogenous H_2_O_2_ from the TME and produce toxic ROS to kill tumor cells. Due to the release of Fe^3+^ in the acidic TME, the tumor overexpressed GSH is consumed, which can protect ROS from consumption while producing Fe^2+^ for CDT. Due to the surface plasmon resonance (SPR) effect, Au NPs can efficiently absorb NIR light and transfer it to PPy to produce more toxic ROS. At the same time, good photothermal conversion performance can promote CDT efficiency. In addition, to maximize the cancer-killing efficiency, the nanoplatform can be successfully loaded with the chemotherapeutic drug DOX, which can be efficiently released under NIR excitation and induction of slight acidity at the tumor site. Both in vitro cell experiments and in vivo tumor-bearing mouse experiments confirmed the nanoplatform’s synergistic multimodal antitumor effect. In conclusion, the proposed TME-mediated Fe_3_O_4_@Au/PPy nanoplatform represents a new and versatile approach for the development of smart nanomedicines in the field of cancer therapy.

## 2. Materials and Methods

### 2.1. Synthesis of Fe_3_O_4_@Au/PPy Nanocomposites

Magnetic Fe_3_O_4_ NPs were prepared according to our previously reported method [37]. First, 1.35 g of FeCl_3_∙6H_2_O was dispersed in 40 mL of ethylene glycol and stirred until it became homogeneous and clear. Then, 3.6 g of NaAc and 1.0 g of polyethylene glycol were added. The mixed solution was stirred vigorously for 30 min and then transferred to a 50 mL Teflon reactor. It was heated at 200 °C for 5 h and cooled naturally to room temperature. Black Fe_3_O_4_ products were recovered and separated by magnet, washed with ultra-pure water and anhydrous ethanol several times, and dried for 8 h at 60 °C.

Au NPs were synthesized as reported previously [38]. After 0.25 mL of HAuCl_4_ solution (0.01 M) was accurately measured, 9.75 mL of CTAB (0.1 M) and 0.6 mL of NaBH_4_ (0.01 M) were mixed with it. The above mixed solution was stirred for 2 min and allowed to stand for 2 h.

The 300 mg of Fe_3_O_4_ was added to 70 mL of DI water and stirred for 15 min to form a dispersion. Subsequently, 15 mL of ethanol solution containing 3 mL of pyrrole was added to the Fe_3_O_4_ dispersion and ultrasonically stirred for 20 min. After that, 15 mL of HCl aqueous solution (6 M) and 10 mL of the obtained Au NPs solution were mixed into the above suspension and ultrasonically stirred for 1.5 h. The black solid obtained by magnet was dispersed in 70 mL of H_2_SO_4_ aqueous solution (0.5 M), with ultrasonic stirring for 4 h. The black suspension was filtered, and the precipitate was washed and dried to obtain the black product, which was labeled as Fe_3_O_4_@Au/PPy. The preparation of Fe_3_O_4_@PPy was similar to that of Fe_3_O_4_@Au/PPy except for the addition of Au NPs.

### 2.2. Photothermal Performance

In order to investigate the photothermal effect, Fe_3_O_4_@PPy or Fe_3_O_4_@Au/PPy nanocomposites with different concentrations (0.1, 0.2 and 0.3 mg/mL) were prepared. The 1 mL dispersion was added to the sample bottle, and the temperature change under the irradiation of 808 nm laser (1 W/cm^2^) was recorded by Fluke Ti32 thermal infrared camera.

### 2.3. Detection of ROS

1,3-Diphenylisobenzofuran (DPBF) was used as a molecular probe to detect ROS production. Typically, 20 mL of DPBF (0.09 mM) was mixed with 1 mL of sample dispersion (300 μg/mL), and the absorbance of DPBF in the solution at 410 nm was measured after 808 nm laser the irradiation at different times. To determine the PDT effect of PPy, we etched the Fe_3_O_4_@PPy with HCl (PPy-Etch) and detected the ROS induced by PPy-Etch.

### 2.4. Consumption of GSH and Generation of OH

After 0.0024 g of Fe_3_O_4_@Au/PPy nanocomposites was added into 5 mL of GSH solution (200 μmol/L) dissolved in N,N-dimethylformamide (DMF) solution, 250 μL of *o*-diphenanthrene solution (100 mmol/L) was added. After stirring for 15 min, the pH of the solution was adjusted to 5.5. The absorbance was measured every hour at a wavelength of 512 nm.

After 0.0028 g of Fe_3_O_4_@Au/PPy nanocomposites was added into 5 mL of GSH solution (200 μmol/L) dissolved in DMF solution, 100 μL of H_2_O_2_ (100 mmol/L) and 0.7 mL of methylene blue (MB) solutions (100 mmol/L) were added and stirred for 15 min. The pH of the solution was adjusted to 5.5. The absorbance at 660 nm was measured every hour at a wavelength of 660 nm.

### 2.5. DOX Loading and Release

In a typical experiment, Fe_3_O_4_@Au/PPy-DOX nanocomposites were prepared by mixing DOX·HCl (at various weights: 1, 3, 5, 10 and 20 mg) in 5 mL of phosphate buffer solution (PBS) with 10 mg Fe_3_O_4_@Au/PPy. The mixture was placed in a dark shaker overnight. Excess DOX was removed by centrifugation, and the precipitate was washed with PBS several times [39,40,41,42,43]. A UV-Vis spectrophotometer was used to measure the absorbance of the collected solution, and the DOX loading on the nanocomposites was calculated by Formula (1) [37,44,45,46]:

(1) $$\mathrm{Loading}\;\mathrm{rate}=\frac{\mathrm{amount}\;\mathrm{of}\;\mathrm{added}\;\mathrm{drug}-\;\mathrm{amount}\;\mathrm{of}\;\mathrm{drug}\;\mathrm{in}\;\mathrm{supermatant}}{\mathrm{amount}\;\mathrm{of}\;\mathrm{added}\;\mathrm{drug}}*100\%\;$$

In the laser-triggered drug release tests, Fe_3_O_4_@Au/PPy-DOX nanocomposites were added into PBS solution at pH 5.0 and 7.4 under 37 °C and irradiated by the 808 nm laser (0.75 W/cm^2^) for different durations. The changes in absorbance of DOX at 490 nm were recorded by UV-Vis spectra. All the tests were performed in triplicate, and the average values were taken to calculate the release ratio of DOX from Fe_3_O_4_@Au/PPy-DOX with time using Formula (2):

(2) $$\mathrm{Loading}\;\mathrm{rate}=\frac{\mathrm{amount}\;\mathrm{of}\;\mathrm{released}\;\mathrm{drug}\;}{\mathrm{amount}\;\mathrm{of}\;\mathrm{loaded}\;\mathrm{drug}}*100\%\;$$

### 2.6. Cell Experiments

The in vitro cytotoxicities of samples were investigated by MTT assay. Firstly, the prepared nanocomposites (1 mg/mL) were dispersed in incubation medium and then diluted to different concentrations (1, 10, 100 μg/mL). HeLa cells were seeded in 96-well plates at a concentration of 5 × 10^4^ cells per well and cultured for 24 h (5% CO_2_, 37 °C) to allow the cells to attach. After removing the incubation medium, 100 μL aliquots of fresh culture medium containing different concentrations of nanocomposites were added to the wells, which were incubated for 4 h. Then, the irradiation groups were exposed to an 808 nm NIR laser at 1 W/cm^2^ for 10 min under the same culture conditions. After all groups were incubated for another 20 h, the cell viability was determined by MTT assay.

To further study the cytotoxicity of the nanocomposites toward HeLa cells, Hoechst 33342 and PI were used to observe the cell-killing situation. Firstly, HeLa cells were seeded in 6-well plates with a density of 5 × 10^4^ cells per well; after adherence, they were incubated with 1 mg/mL of Fe_3_O_4_@Au/PPy or Fe_3_O_4_@Au/PPy-DOX with or without a magnetic field for about 0.5 h. For phototherapy, corresponding culture dishes were further incubated for 1 h followed by exposure to NIR laser light at 808 nm for 10 min. After irradiation, the HeLa cells were incubated again for 12 h (5% CO_2_, 37 °C), followed by washing with PBS (pH = 7.4) to remove the culture medium and staining with 0.5 mL of Hoechst 33342 (1 μg/mL) or PI (1 μg/mL) for 10 min under darkness. The viable cells or necrotic cells could be identified by intact nuclei dyed with Hoechst 33342 or PI, respectively. Dual fluorescence-stained culture media were washed, and the cells were observed and imaged using an inverted fluorescence microscope. All experiments were performed in triplicate.

### 2.7. Animal Models

Mice were obtained from the Laboratory Animal Center of Anhui Medical University (Certification of Quality 34000200000077, 34000200000078). This study was conducted in strict accordance with the recommendations in the Regulations on the Management of Laboratory Animals in China promulgated in 1988. All the animal experiments were in agreement with the guidelines of the Animal Experiments and Care Regulations of Anhui University and approved by the Animal Use Committee of Anhui University.

### 2.8. In Vivo Synergistic Antitumor Effect

H22 cells were suspended in 100 μL of PBS and injected into the back of the hind leg of each mouse. The mice were divided into nine groups: (1) PBS, (2) DOX, (3) DOX+L, (4) Fe_3_O_4_@PPy, (5) Fe_3_O_4_@PPy+L, (6) Fe_3_O_4_@Au/PPy, (7) Fe_3_O_4_@Au/PPy+L, (8) Fe_3_O_4_@Au/PPy-DOX and (9) Fe_3_O_4_@Au/PPy-DOX+L. Each group was treated with different samples which were injected via tail vein at the dose of 5–6 mg/kg (sample/mouse weight) and then exposed to different external conditions. Afterward, the mice were weighed and the tumor sizes were measured by a caliper every day; the tumor volume was calculated by Formula (3), and the tumor inhibition rate was calculated by Formula (4):

(3) $$\mathrm{Tumor}\;\mathrm{volume}\;\left({\mathrm{mm}}^{3}\right)=\frac{ab^{2}}{2}$$

(4) $$\mathrm{Tumor}\;\mathrm{inhibition}\;\mathrm{rate}\left(\%\right)=1-\frac{V_{1}}{V_{2}}\times 100\%$$

where *a* and *b* mean the maximum length (mm) and minimum width (mm) of the tumor, respectively. After the treatment of 18 days, all the mice were sacrificed, the tumor of each mouse was collected and cleaned and digital photos of tumors were obtained using a camera.

## 3. Results and Discussion

**Figure 1:**
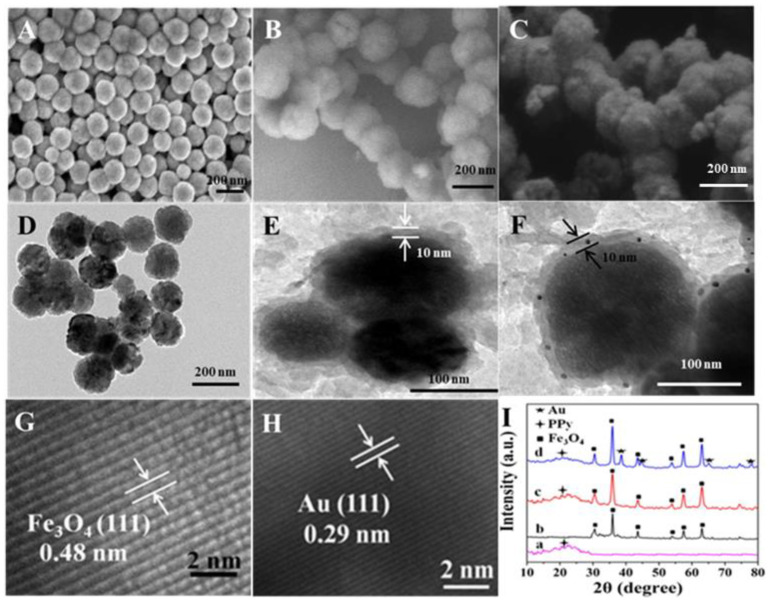
(**A**–**C**) SEM and (**D**–**F**) TEM images of (**A**,**D**) Fe_3_O_4_, (**B**,**E**) Fe_3_O_4_@PPy and (**C**,**F**) Fe_3_O_4_@Au/PPy. (**G**,**H**) HRTEM images of Fe_3_O_4_@Au/PPy nanocomposites. (**I**) XRD patterns of (a) PPy, (b) Fe_3_O_4_, (c) Fe_3_O_4_@PPy and (d) Fe_3_O_4_@Au/PPy. Fe_3_O_4_, PPy and Au diffraction peaks are labeled as “■”, “✦” and “★”, respectively.

A novel self-sacrificial template route was proposed for the synthesis of Fe_3_O_4_@Au/PPy nanocomposites, and Fe_3_O_4_ and Fe_3_O_4_@PPy were also successfully synthesized for the control experiment. Figure 1 shows the microstructure and phase composition of Fe_3_O_4_, Fe_3_O_4_@PPy and Fe_3_O_4_@Au/PPy NPs. A typical scanning electron microscope (SEM) image (Figure 1A) and a typical transmission electron microscopy (TEM) image (Figure 1D) indicated that the obtained Fe_3_O_4_ NPs exhibited a uniform spherical shape with a diameter of about 150 nm. Fe_3_O_4_@PPy and Fe_3_O_4_@Au/PPy nanocomposites also had uniform sphere-like structures with diameters larger than Fe_3_O_4_ NPs, which may be attributed to the successful capping of PPy on the surface of Fe_3_O_4_ NPs (Figure 1B,C). It can be determined from Figure 1E that the Fe_3_O_4_ core was covered with a PPy layer with a thickness of about 10 nm, which presented a clear core–shell structure. As shown in Figure 1F, Au NPs were inserted into the PPy shell. The HRTEM images of Fe_3_O_4_@Au/PPy nanocomposites (Figure 1G) indicated a lattice spacing of 0.48 nm, which is attributed to the (110) plane of Fe_3_O_4_, demonstrating the presence of a Fe_3_O_4_ core. After Au NPs were added in the synthesis process, a small number of black spots could be observed in the PPy shell layer in the TEM image (Figure 1F), suggesting the existence of Au NPs and the successful preparation of Fe_3_O_4_@Au/PPy nanocomposites. When high-resolution transmission electron microscopy (HRTEM) images of the shell region were taken, lattice fringes of 4.80 and 2.90 Å were observed, which corresponded to the (110) planes of Fe_3_O_4_ and the (111) plane of Au, respectively. Subsequently, the composition and crystalline structure of the products were characterized by X-ray diffraction (XRD) patterns (Figure 1I). In Figure 1I-a, there is only a broad peak located at 2*θ* of 20–30°, which implies that pure PPy was amorphous [34]. The characteristic diffraction peaks of Fe_3_O_4_ appeared at 2*θ* of 30.40°, 35.42°, 43.20°, 53.40°, 57.20° and 62.70° (Figure 1I-b), which were well matched with the (220), (311), (400), (422), (511) and (440) planes of Fe_3_O_4_ (JCPDS No. 19-0629), respectively. Meanwhile, it can be clearly seen from Figure 1I-c that there was not only the broad peak of PPy but also the diffraction peaks of Fe_3_O_4_ in the Fe_3_O_4_@PPy nanocomposites, indicating the formation of Fe_3_O_4_@PPy nanocomposites. In Figure 1I-d, besides the diffraction peaks of PPy and Fe_3_O_4_ in Fe_3_O_4_@Au/PPy nanocomposites, there are four new peaks at 2*θ* of 38.5°, 44.6°, 64.8° and 77.7°, representing the (111), (200), (220) and (311) planes of Au (JCPDS card No. 04-0784), which demonstrates the successful embedding of Au NPs into Fe_3_O_4_@PPy nanocomposites. This evidence indicated that Fe_3_O_4_@Au/PPy nanocomposites were successfully synthesized by the sacrificial template method.

**Figure 2:**
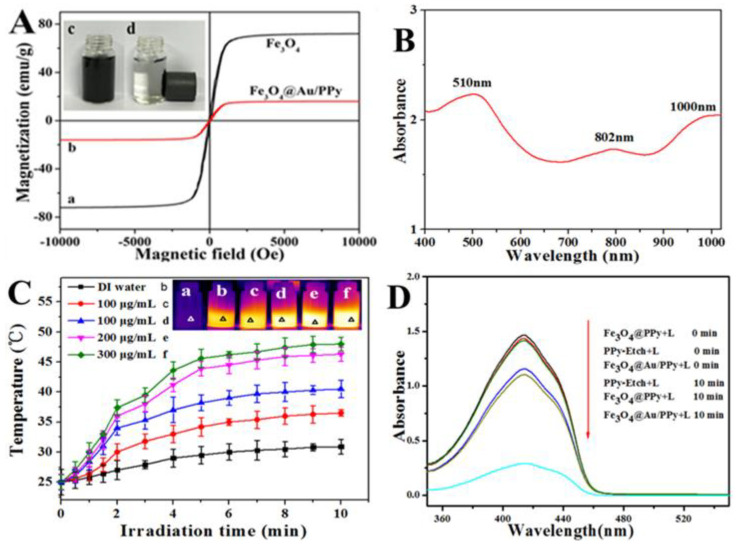
(**A**) Magnetization curves of (a) Fe_3_O_4_ and (b) Fe_3_O_4_@Au/PPy nanocomposites. The top left inset presents photographs of the Fe_3_O_4_@Au/PPy aqueous dispersion (c) before and (d) after magnetic separation by an external magnetic field. (**B**) UV-Vis-NIR spectrum of Fe_3_O_4_@Au/PPy. (**C**) Temperature change curves and thermal images (inset) of (b) the DI water; (c) Fe_3_O_4_@PPy at 100 μg/mL; Fe_3_O_4_@Au/PPy at (d) 100 μg/mL, (e) 200 μg/mL and (f) 300 μg/mL irradiated with 808 nm laser at 1 W/cm^2^ for 10 min. In the inset, (a) shows the thermal image of the DI water at room temperature (25 °C). Error bars are based on at least triplicate measurements. (**D**) Absorbance changes at 410 nm of DPBF solutions in the presence of Fe_3_O_4_@PPy, PPy-Etch and Fe_3_O_4_@Au/PPy with or without irradiation (808 nm). *L* represents light irradiation.

Due to the presence of Fe, the magnetic hysteresis loops of Fe_3_O_4_ NPs and Fe_3_O_4_@Au/PPy nanocomposites were investigated by applying an external magnetic field. As shown in Figure 2A, the saturation magnetization of Fe_3_O_4_ NPs was 68 emu/g, while that of Fe_3_O_4_@Au/PPy nanocomposites was 20 emu/g. Weakening of the magnetization of nanocomposites may be ascribed to the presence of the non-magnetic PPy shell and embedded Au NPs. The illustration at the top left of Figure 2A exhibits that the nanocomposites in water were attracted toward the external magnetic field, revealing the prominent magnetic property of the Fe_3_O_4_@Au/PPy nanocomposites. The hysteresis loop diagram of the Fe_3_O_4_@Au/PPy nanocomposite in the low magnetic field region (Figure S1, in the Supporting Materials) demonstrates the ferromagnetic state. The above results indicated Fe_3_O_4_@Au/PPy nanocomposites have good application prospects for magnetic-targeted drug delivery. Figure 2B shows the UV-Vis-NIR spectroscopy results exhibiting the optical property of the resultant Fe_3_O_4_@Au/PPy. The visible-to-NIR region had an intense and broad absorption band, and three absorption peaks of the nanocomposites appeared at around 510 nm, 802 nm and 1000 nm, which may have resulted from the polaron and bipolaron band transitions for PPy and the plasmon resonance absorption of Au [37], indicating that the nanocomposites could be used for photothermal therapy by transforming the absorbing NIR light energy into heat.

Figure 2C shows the photothermal curves of DI water, Fe_3_O_4_@PPy and Fe_3_O_4_@Au/PPy aqueous dispersions with different concentrations under NIR irradiation from 0 to 10 min. It can be clearly observed that Fe_3_O_4_@Au/PPy nanocomposites had a better photothermal effect than Fe_3_O_4_@PPy at the same concentration, indicating the presence of Au NPs enhanced the photothermal efficiency. In addition, the concentration of Fe_3_O_4_@Au/PPy was positively correlated with the photothermal effect. The temperature enhancement of Fe_3_O_4_@Au/PPy nanocomposite dispersion (300 μg/mL) could reach about 22.5 °C after 10 min of irradiation. The photothermal impact of the products was further vividly demonstrated by the thermal images (inset of Figure 2C). These results illustrated that the combination of PPy and Au NPs could achieve a synergistic effect for photothermal therapy.

The photodynamic effects of Fe_3_O_4_@PPy, PPy-Etch and Fe_3_O_4_@Au/PPy were investigated by using DPBF as an indicator. The measured peaks of DPBF solutions at 410 nm over different samples with or without NIR light are presented in Figure 2D. For all samples, the absorption peak intensity at 410 nm was weakened after irradiation, showing all three samples had a PDT effect. Compared with Fe_3_O_4_@PPy and PPy-Etch, it was found that not only the PPy but also Fe_3_O_4_ NPs could have a PDT effect after irradiation with an 808 nm laser for 10 min. After 10 min of irradiation, the PDT effect of Fe_3_O_4_@Au/PPy with embedded Au NPs was significantly enhanced compared to Fe_3_O_4_@PPy, which was attributed to the fluorescence resonance energy transfer (FRET) process between Au NPs and PPy. Au NPs could absorb the NIR light efficiently because of the surface plasmon resonance (SPR) effect [47] and transfer the absorbed light energy to PPy for generating more ROS. The above results showed that the Fe_3_O_4_@Au/PPy nanocomposites can be used as an effective photodynamic agent for inducing the generation of ROS and killing tumor cells.

**Figure 3:**
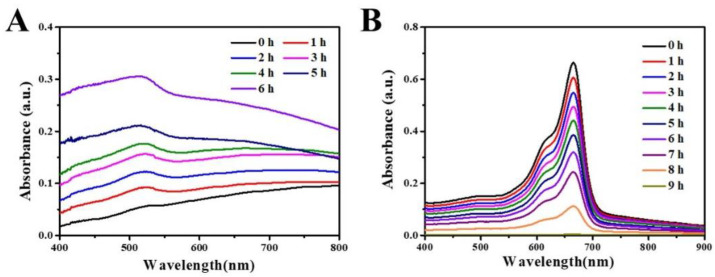
Degradation curves of (**A**) *o*-diphenanthrene and (**B**) MB by Fe_3_O_4_@Au/PPy dispersions at various times (h).

As we know, Fe_3_O_4_ in the prepared nanocomposite could produce Fe^3+^ ions in the slightly acidic environment of tumors. Then Fe^3+^ ions can be converted to Fe^2+^ ions with expressed GSH on the tumor cell membrane (Chemical Equation (5)). The *o*-diphenanthrene can react with Fe^2+^ ions to form a complex and exhibit a characteristic absorption peak at 512 nm. Therefore, the consumption of GSH and generation of Fe^2+^ ions were verified by UV-Vis spectroscopy. As seen in Figure 3A, the absorbance of the mixed solution at 512 nm gradually increases with time, indicating that Fe^3+^ ions played important roles in the oxidation of GSH and the effective release of Fe^2+^ ions. Thus, GSH was consumed in large quantities, effectively promoting the photodynamic effect.

Fe^3+^ + GSH → Fe^2+^ + GSSG (5)

In addition, Fe^2+^ ions generated under acidic conditions and from the GSH consumption can further react with H_2_O_2_ to produce ·OH through the Fenton reaction to achieve CDT (Chemical Equation (6)). The generation of ·OH was confirmed by the degradation of methylene blue (MB). As shown in Figure 3B, MB was almost completely degraded in the Fe_3_O_4_@Au/PPy dispersion after a reaction of 9 h. Therefore, we can conclude that Fe_3_O_4_@Au/PPy not only has multiple responses to the tumor environment, such as weak acid, endogenous hydrogen peroxide and GSH, but also regulates and improves the tumor microenvironment. These results indicated that Fe_3_O_4_@Au/PPy nanocomposites can induce the formation of ·OH to realize CDT, as well as promote PDT and CDT effects by consuming GSH, thus exerting a synergistic antitumor effect.

Fe^2+^ + H_2_O_2_ → 2Fe^3+^ + OH^−^ +·OH (6)

**Figure 4:**
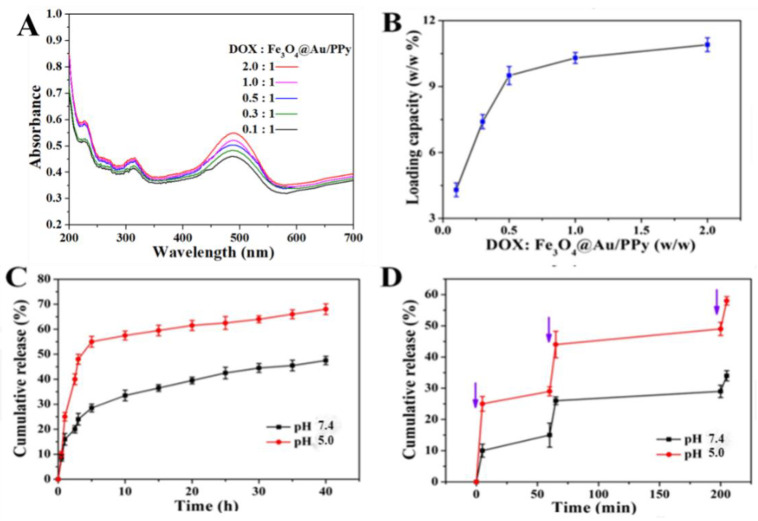
(**A**) UV-Vis absorption spectra of Fe_3_O_4_@Au/PPy-DOX dispersions with different weight ratios of DOX to Fe_3_O_4_@Au/PPy. (**B**) Quantitative analysis of DOX loading at different weight ratios of DOX to Fe_3_O_4_@Au/PPy. (**C**) In vitro drug release behavior of Fe_3_O_4_@Au/PPy-DOX in PBS at pH 7.4 and 5.0. (**D**) In vitro cumulative drug release from Fe_3_O_4_@Au/PPy-DOX. The samples at pH 7.4 and 5.0 were irradiated with a NIR laser (1 W/cm^2^) for 5 min at different time points indicated by the violet arrows. The weight ratio of DOX to Fe_3_O_4_@Au/PPy is 2.0:1 in Figure 5A,B.

Fe_3_O_4_@Au/PPy nanoplatform not only had the multifunctional effects of PTT, CDT and PDT, but its PPy shell also offered the possibility of loading drugs. The anticancer drug DOX was loaded into the Fe_3_O_4_@Au/PPy nanoplatform and its drug release behavior was recorded. Figure 4A shows the UV-Vis absorption spectrum of Fe_3_O_4_@Au/PPy-DOX; the characteristic peak at 490 nm can be attributed to DOX, indicating the successful loading. With the increasing proportion of DOX to Fe_3_O_4_@Au/PPy, the intensity of the DOX characteristic peak was gradually enhanced. The strength was greatest when the weight ratio was 2:1. The results were consistent with the quantitative analysis shown in Figure 4B. It can be clearly seen that the loading efficiency of DOX shows an upward trend with the addition of DOX, and the saturation loading rate was about 11%, which was not objective compared with other literature [22,23]. Subsequently, the release behavior of Fe_3_O_4_@Au/PPy-DOX at different pH values was tracked, as shown in Figure 4C. It can be found that the release rate of DOX was 69% at pH 5.0, while the release rate was 47% at pH 7.4. This may be due to protonation of the amino group in DOX, which provided DOX with a positive charge and facilitated drug release at acidic pH. Furthermore, by controlling the switch of NIR, a slow and controlled DOX release behavior of the Fe_3_O_4_@Au/PPy-DOX nanoplatform can be achieved. According to Figure 4D, the cumulative DOX release amount of the Fe_3_O_4_@Au/PPy-DOX nanoplatform increased rapidly each time under NIR laser irradiation. The results showed that the release process of NIR light may be due to the loose PPy shell caused by the rapid rise in local temperature. Notably, drug release was more significant at acidic pH 5.0, while DOX release was limited at acidic pH 7.4. This special property provided the basis for the specific release of DOX in the acidic environment of the tumor.

**Figure 5:**
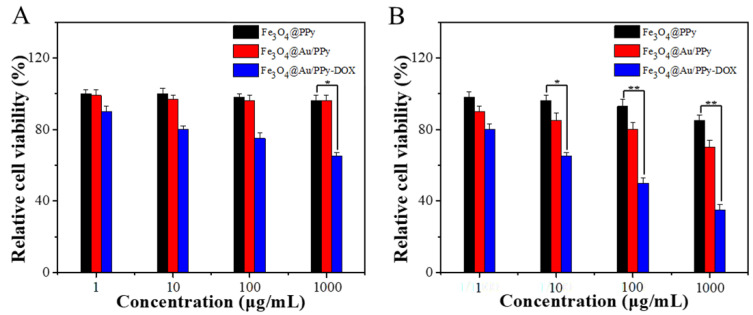
Viability of HeLa cells incubated with different concentrations of Fe_3_O_4_@PPy, Fe_3_O_4_@Au/PPy and Fe_3_O_4_@Au/PPy-DOX (the weight ratio of DOX to Fe_3_O_4_@Au/PPy was 2.0:1) (**A**) without and (**B**) with irradiation. Means ± SD (n = 3). * *p* < 0.5, ** *p* < 0.01.

Based on the excellent properties of the Fe_3_O_4_@Au/PPy-DOX nanoplatform, it was expected to become a novel anticancer agent. The relative viability of HeLa cells and different concentrations of Fe_3_O_4_@PPy, Fe_3_O_4_@Au/PPy and Fe_3_O_4_@Au/PPy-DOX after incubation under and without laser irradiation were measured by the MTT method. As expected from Figure 5A, Fe_3_O_4_@PPy and Fe_3_O_4_@Au/PPy exhibited a negligible cytotoxicity, even at 1 mg/mL. The co-presence of Fe_3_O_4_@Au/PPy and 808 nm laser irradiation reduced cell viability, suggesting that the generated heat and ROS of Fe_3_O_4_@Au/PPy under the NIR laser irradiation could efficiently kill tumor cells (Figure 5B). In contrast, with the laser irradiation, the viability of cells incubated with Fe_3_O_4_@Au/PPy-DOX was lower (35%), indicating the synergetic anticancer effect from Fe_3_O_4_@Au/PPy as well as the anticancer drug DOX.

**Figure 6:**
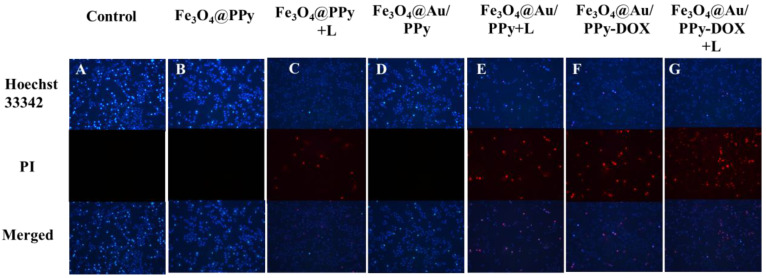
Fluorescence microscopy images of HeLa cells incubated with (**A**) nothing, (**B**,**C**) Fe_3_O_4_@PPy, (**D**,**E**) Fe_3_O_4_@Au/PPy and (**F**,**G**) Fe_3_O_4_@Au/PPy-DOX (the weight ratio of DOX to Fe_3_O_4_@Au/PPy is 2.0:1) with or without NIR laser irradiation. L represent NIR laser. Shown are HeLa cells dyed blue by Hoechst 33342 and red by PI and the merged images of both.

Fluorescence images were used to further demonstrate the antitumor effect of Fe_3_O_4_@PPy, Fe_3_O_4_@Au/PPy and Fe_3_O_4_@Au/PPy-DOX in vitro. As shown in Figure 6B,D, the death of HeLa cells incubated with Fe_3_O_4_@PPy or Fe_3_O_4_@Au/PPy in the absence of irradiation was almost not observed (essentially no red fluorescence) owing to the good biocompatibility and low cytotoxicity of these two nanoparticles. However, when HeLa cells were cultured with Fe_3_O_4_@PPy or Fe_3_O_4_@Au/PPy with laser irradiation (Figure 6C,E), the fluorescence image showed a merge of red and purple, revealing the killing effects of HeLa cells by PDT and PTT from nanocomposites. As shown in Figure 6F, the cells cultured with Fe_3_O_4_@Au/PPy-DOX without laser irradiation were partially killed, which may be due to the slightly acidic environment of tumor cells leading to a partial release of DOX. Strong red fluorescence could be seen after irradiation of HeLa cells cultured with Fe_3_O_4_@Au/PPy-DOX (Figure 6G), indicating that Fe_3_O_4_@Au/PPy-DOX had an obvious antitumor effect of CT combined with multiple treatments under the excitation of NIR light, which was consistent with the MTT results.

**Figure 7:**
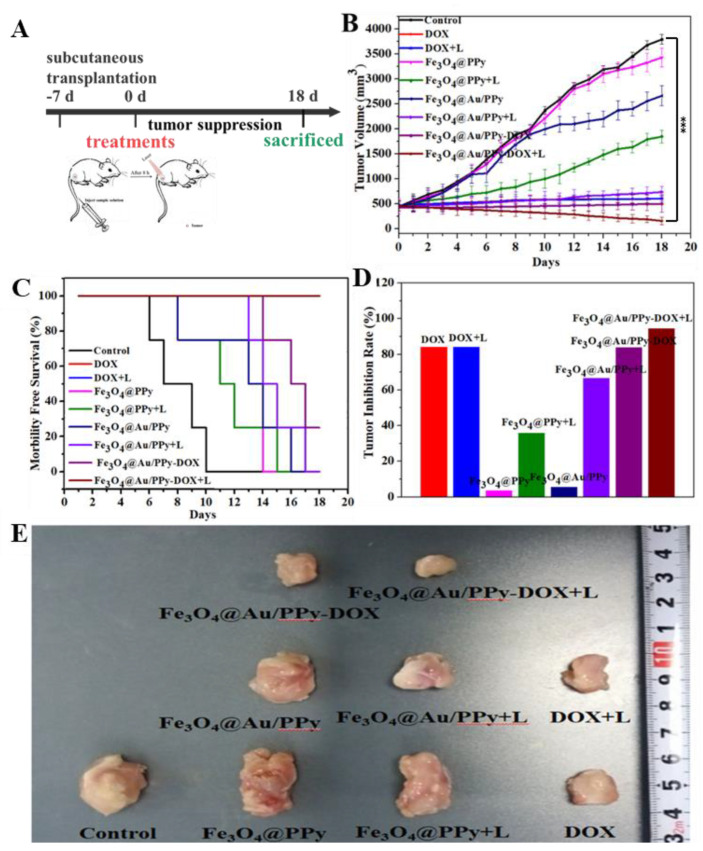
In vivo synergistic antitumor effect of products. (**A**) Schematic diagram of the samples in vivo anticancer therapy. (**B**) Tumor volume curves, (**C**) survival curves, (**D**) tumor inhibition rate and (**E**) representative tumor photos of H22 tumor-bearing mice after different treatments. Means ± SD (n = 5). *** *p* < 0.001.

Although Fe_3_O_4_@Au/PPy-DOX had a good therapeutic effect in vitro, its therapeutic effect in vivo also deserved to be further explored (Figure 7A). We selected H22 tumor-bearing mice as a model and randomly divided them into nine groups: (1) PBS, (2) DOX, (3) DOX+L, (4) Fe_3_O_4_@PPy, (5) Fe_3_O_4_@PPy+L, (6) Fe_3_O_4_@Au/PPy, (7) Fe_3_O_4_@Au/PPy+L, (8) Fe_3_O_4_@Au/PPy-DOX and (9) Fe_3_O_4_@Au/PPy-DOX+L. Figure 7B shows the tumor volume changes of H22 tumor-bearing mice after different treatments. Compared with other groups, the group of Fe_3_O_4_@Au/PPy-DOX with light irradiation was found to exhibit the best suppression, which demonstrated the excellent synergistic antitumor effect of Fe_3_O_4_@Au/PPy-DOX under NIR light. The body weight of each treatment group increased slightly as a result of the normal feeding process (Figure S2). Overall, there was no significant difference in body weight in each group after treatment, indicating that the prepared samples had high therapeutic biosafety. The excellent antitumor effect was further proved by Figure 7C–E. In Figure 7C, we can observe that the Fe_3_O_4_@Au/PPy-DOX group possessed an outstanding ability to inhibit tumor growth under NIR light; the tumor inhibition rate could reach up to 91% (Figure 7D). The digital photo in Figure 7E intuitively shows the size of each group of tumors. Compared with other groups, the tumor growth of H22 tumor-bearing mice injected with Fe_3_O_4_@Au/PPy-DOX with light irradiation was significantly inhibited. The in vivo experiment vividly certified that Fe_3_O_4_@Au/PPy-DOX could serve as a preferred nanomaterial for trimodal antitumor therapy. These in vivo experiments vividly demonstrated that Fe_3_O_4_@Au/PPy-DOX may be an excellent antitumor nanoagent combined with multiple therapeutic approaches under the action of NIR light and the tumor microenvironment.

## 4. Conclusions

In summary, in this work, a magnetic-targeted Fe_3_O_4_@Au/PPy-DOX nanoplatform with TME-mediated and NIR-excited multimodel tumor therapy was developed by following a sacrificial template strategy. When Fe_3_O_4_@Au/PPy-DOX is aggregated at the tumor site, it can produce heat and ROS under NIR light for PTT and PDT. Under the stimulation of the acidic environment and GSH overexpression in a tumor, Fe_3_O_4_@Au/PPy-DOX can release Fe^3+^ to catalyze the overexpressed H_2_O_2_ to generate ·OH through the Fenton reaction, which can be used to realize CDT and increase ROS production. Simultaneously, the low-pH environment of the tumor can jointly trigger the release of DOX in the nanoplatform to achieve an effective synergistic antitumor therapy. It was confirmed that this work will provide distinctive horizons for the development of more novel multifunctional antitumor nanoplatforms. Meanwhile, nanoplatforms with more triggering mechanisms and more DOX capacity are expected to be investigated in the future.

## Supplementary Materials

The following supporting information can be downloaded at: https://www.mdpi.com/article/10.3390/molecules27134003/s1, Figure S1: The enlarged version of Figure 2A in the region of low magnetic field.
