# Supplementary material for: Facile Synthesis of Fe3O4@Au/PPy-DOX Nanoplatform with Enhanced Glutathione Depletion and Controllable Drug Delivery for Enhanced Cancer Therapeutic Efficacy
Source: Molecules. 2022 Jun 22;27(13):4003. doi: 10.3390/molecules27134003 (PMC9268512; doi:10.3390/molecules27134003)
Supplement: Supplementary file 1 [file molecules-27-04003-s001.zip › molecules-1740894-supplementary.pdf]

Supporting Information

NIR-driven Fe_3O_4@Au/PPy-DOX as the tumor microenvironment-mediated multifunctional nanoplatform for enhanced cancer therapy

Chunxia Qi[a,b,#], Wanni Wang[c,#], Peisan Wang[c,*], Hanlong Cheng[a], Xueyan Wang[a], Baoyou Gong[a], Anjie Xie[a], Yuhua Shen[a,*]

a College of Chemistry and Chemical Engineering, Anhui University, Hefei 230601, P. R. China.

b Department of Chemical Engineering, Hefei Normal University, Hefei 230601, P. R.China

c School of Biomedical Engineering, Research and Engineering Center of Biomedical Materials, School of Basic Medical Sciences Anhui Medical University, Hefei 230032, P. R. China.

***Corresponding author**

Dr. Peisan Wang: wangpeisan@ahmu.edu.cn

Prof. Yuhua Shen: s_yuhua@163.com

Materials and methods

Materials

Iron trichloride hexahydrate ($FeCl_3 \cdot 6H_2O$), ethylene glycol (EG, 98%), polyethylene glycol (PEG, MW=2000), sodium acetate (CH_3COONa, 99.0%), gold chloride tetrahydrate ($HAuCl_4 \cdot 4H_2O$), pyrrole, hydrochloric acid (HCl), hydrogen peroxide (H_2O_2), Glutathione (GSH, reduced) ($C_{10}H_{17}N_3O_6S$), methylene blue (MB) and concentrated sulfuric acid (H_2SO_4) were obtained from Aladdin Reagent Co., Ltd. (Shanghai, P. R. China). Dulbecco's Modified Eagle's Medium (DMEM), fetal calf serum, trypsin-EDTA solution, 3-[4, 5-dimethylthialzol-2-yl]-2, 5-diphenyltetrazolium bromide (MTT), Diphenylbenzofuran (DPBF), Hoechst 33342, propidium iodide (PI), and doxorubicin hydrochloride (DOX·HCl) were purchased from Tianjin Chemical Co., Ltd. (P. R. China). HeLa cells, immortal human cervical cancer cells used in scientific research and derived from Henrietta Lacks, were obtained from the Key Laboratory of Ecological Engineering and Biotechnology of Anhui Province, School of Life Science, Anhui University (Hefei, P. R. China). Female ICR mice were purchased from the Laboratory Animal Center of AnHui Medical University. All chemical reagents used in this experiment were of analytical grade without further purification. Deionized (DI) water is obtained from Millipore Milli-Q system (resistivity: 18.2 MΩ.cm).

Characterization

Transmission electron microscopy (TEM) images were obtained using a JEM-100SX instrument. Scanning electron microscope (SEM) was performed on an S-4800 (Hitachi Co., Ltd., Japan) scanning electron microscope. Ultraviolet Visible (UV-Vis) measurements were performed by an UV-3900 spectrometer (Hitachi Co., Ltd., Japan). X-ray diffraction (XRD) patterns were recorded using a DX-2700 diffractometer (Dandong Fangyuan Instrument Co. Ltd., P. R. China). A superconducting quantum interference device (SQUID) magnetometer (Quantum Design PPMS XL-7) was used to measure the magnetic properties of the as-prepared samples. The OD values of the MTT assay were measured by a RT-2100C spectro-photometric micro-plate reader (Rayto, Shenzhen, P. R. China). Fluorescence microscopy images were obtained using

a DMI3000B inverted fluorescence microscope (Leica, Germany).

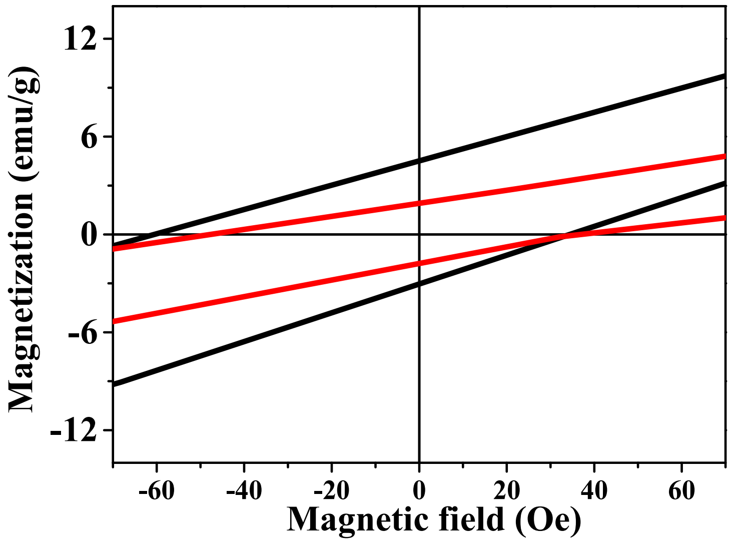

Figure S1 The enlarged version of Figure 2A in the region of low magnetic field.
